# Regional lymph node density-based nomogram predicts prognosis in nasopharyngeal carcinoma patients without distant metastases

**DOI:** 10.1186/s40644-023-00641-z

**Published:** 2023-12-15

**Authors:** Jie Ma, Rong Zhao, Yu-Lan Wu, Yang Liu, Guan-Qiao Jin, Dan-Ke Su

**Affiliations:** 1 Medical Imaging Department, Guangxi Medical University Cancer Hospital, Guangxi, China; 2 Department of Radiation Oncology, Guangxi Medical University Cancer Hospital, Guangxi, China

**Keywords:** Nasopharyngeal carcinoma, Lymph node, Magnetic resonance imaging, Prognosis

## Abstract

**Background:**

Nasopharyngeal carcinoma (NPC) is a relatively common type of cancer in Southern China, with local recurrence or distant metastases even after radical treatment; consequently, it is critical to identify the patients at higher risk for these events beforehand. This study aimed to assess the prognostic value of regional lymph node density (RLND) associated nomograms in NPC and to evaluate the utility of nomograms in risk stratification.

**Methods:**

A total of 610 NPC patients without distant metastases (425 in the training and 185 in the validation cohort) were enrolled. The MRI-identified nodal features and clinical characteristics were documented, and the RLND was calculated. Cox analyses were conducted to identify prognostic-associated factors. Nomograms were generated based on the multivariate analysis results. The predictive accuracy and discriminative ability of the nomogram models were determined using the concordance index (C-index), receiver operating characteristic (ROC) curve, and calibration curve; the results were compared with those of the tumor-node-metastasis (TNM) classification. Decision curve analysis (DCA) and C-index were used to assess the prognostic effect and added discriminative ability of RLND. We also estimated the optimal RLND-based nomogram score cut-off values for survival prediction.

**Results:**

RLND was an independent predictor of overall survival (OS) and disease-free survival (DFS), with hazard ratios of 1.36 and 1.30, respectively. RLND was utilized in the construction of nomograms, alongside other independent prognostic factors. The RLND-based nomogram models presented a more effective discriminative ability than the TNM classification for predicting OS (C-index, 0.711 vs. 0.680) and DFS (C-index, 0.681 vs. 0.669), with favorable calibration and consistency. The comparison of C-index values between the nomogram models with and without RLND provided substantiation of the crucial role RLND plays in these models. DCA confirmed the satisfactory clinical practicability of RLND. Moreover, the nomograms were used to categorize the patients into three groups (high-, middle-, and low-risk), and the Kaplan–Meier curves showed significant differences in prognosis between them (*p* < 0.05). These results were verified in the validation cohort.

**Conclusion:**

RLND stands as a robust prognostic factor in NPC. The RLND-based nomograms excel in predicting survival, surpassing the TNM classification.

**Supplementary Information:**

The online version contains supplementary material available at 10.1186/s40644-023-00641-z.

## Background

Nasopharyngeal carcinoma (NPC) is a head and neck malignancy with a relatively high incidence in Southern China and Southeast Asia [1]. Radiotherapy-based strategies with or without systemic therapy are the mainstay of treatment for NPC patients without distant metastases [2, 3], and the 5-year overall survival (OS) has exceeded 80%, thanks to the considerable progress in multidisciplinary treatment and radiotherapy techniques [4, 5]. However, local recurrence or distant metastases still commonly occur after radical treatment, particularly in patients with stage II-IVa NPC. When local recurrences or distant metastases occur, it significantly affects the prognosis of patients. Therefore, accurately and early identifying patients at high risk for a poor prognosis and initiating early interventions that may prolong their survival are critical challenges for physicians [6].

The 8th edition of the tumor node metastasis (TNM) classification system from the American Joint Committee on Cancer (AJCC) is the gold standard for evaluating the disease status and prognosis in patients with NPC [7, 8]. However, this classification system shows a limited predictive capacity and is insufficient to meet the increasing clinical needs for individualized treatment [9–11]. Therefore, it is crucial to identify robust markers to assist in risk stratification and facilitate the development of a model to accurately predict survival in these patients.

Previous studies have suggested that several hematological biomarkers are related to survival in NPC [12, 13]. Nonetheless, incorporating additional nodal characteristics into the current classification system could improve the accuracy of survival prediction [14–16]. However, the most reliable and robust predictor remains to be identified. The location of lymph nodes affected by the disease is an important stratification factor in N classification [7, 8, 17], and the number of positive lymph nodes (pLNs) reportedly has a considerable prognostic value in NPC [18, 19]. A combination of these two factors may create a robust predictive marker. Regional lymph node density (RLND) is defined as the ratio of the number of pLNs to the number of lymphatic drainage regions involved; it quantifies the lymph node burden more comprehensively and may be closely related to NPC prognosis.

The prognostic value of RLND remains unclear in patients with NPC. The objective of this study was to evaluate the prognostic significance of RLND-based nomograms in NPC and assess the effectiveness of nomograms in stratifying patient risk.

## Methods

**Fig. 1:**
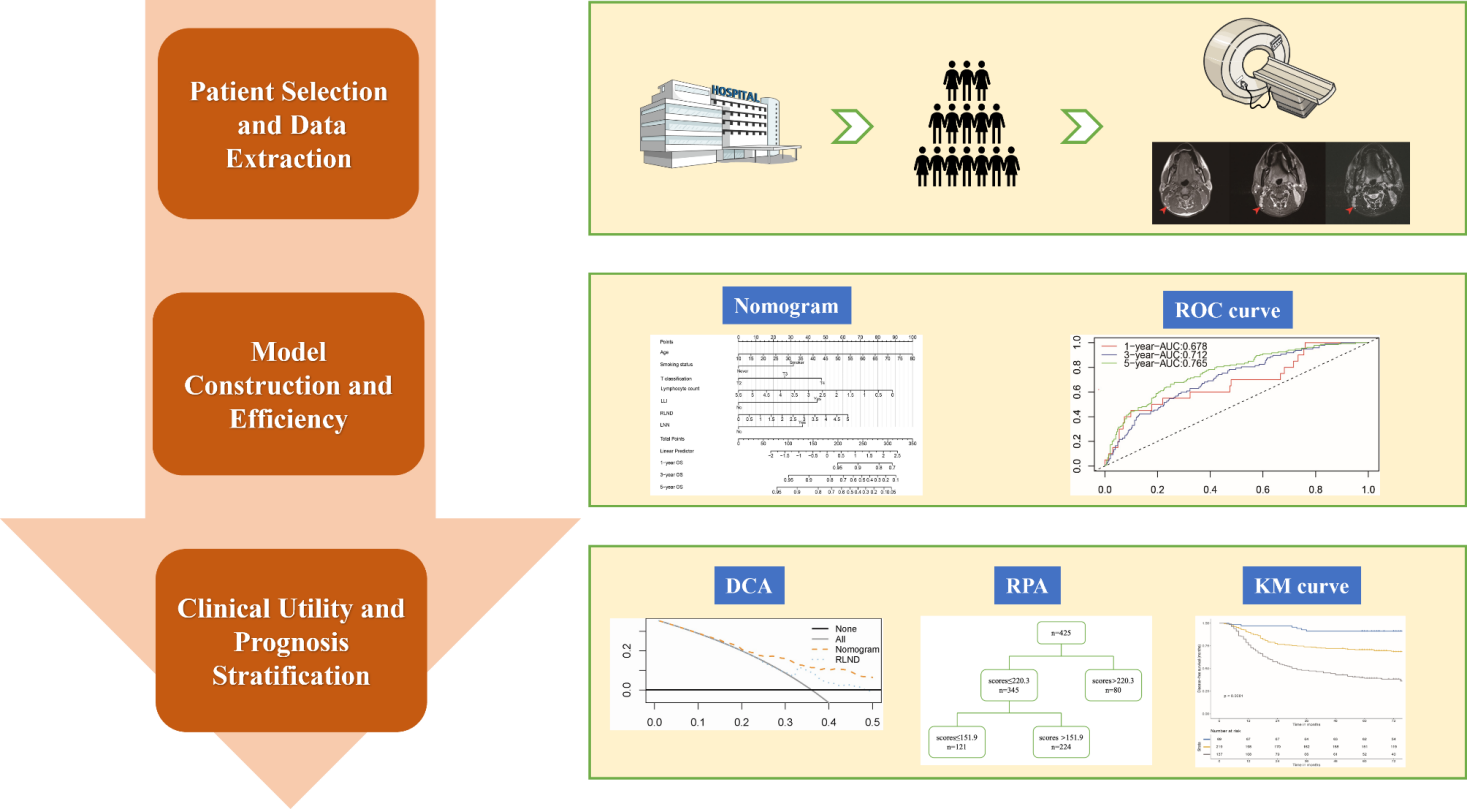
Flowchart illustrates the study design

This study was approved by the institutional review board (IRB) of Guangxi Medical University Cancer Hospital. The requirement for written informed consent was waived due to its retrospective nature; the study design is illustrated in Fig. 1.

### Study Population

Newly diagnosed patients with NPC were identified in Guangxi Medical University Cancer Hospital between October 2014 and December 2017. All patients underwent routine evaluations including history taking, physical examination, hematology and biochemistry profiling, fiberoptic nasopharyngoscopy, neck and nasopharyngeal MRI, chest and abdominal computed tomography (CT), skeletal scintigraphy and/or positron emission tomography/computed tomography (PET/CT) for assessing general conditions. The inclusion criteria were as follows: (1) biopsy-confirmed NPC; (2) stage II-IVa according to the 8th AJCC staging system; (3) magnetic resonance imaging (MRI) scan of the neck and nasopharyngeal area at the initial diagnosis; (4) treatment with intensity-modulated radiotherapy (IMRT); and (5) complete imaging, clinical, and follow-up data. Exclusion criteria were synchronous malignancies, pregnancy or breastfeeding, and uncontrolled cardiac, pulmonary, renal, or liver diseases. In total, 610 patients were included in this study; 425 patients were considered the training cohort from October 2014 to December 2016, and 185 patients as the validation cohort from January 2017 to December 2017.

### Collection of pretreatment data

Clinical data were collected from the medical records. The hematological parameters of patients were collected within one week before commencing treatment. They included Epstein–Barr virus (EBV) DNA, white blood cell count, hemoglobin, platelet count, neutrophil count, monocyte count, lymphocyte count, albumin, alkaline phosphatase, and lactate dehydrogenase.

### MRI Acquisition

All patients underwent pretreatment MR imaging for primary tumor staging at the initial diagnosis. MR imaging examinations were performed at 1.5 T (Magnetom Avanto, Siemens Healthcare, Erlangen, Germany) by using a head and neck coil. The acquisition sequences included: (1) Scanning: cross-sectional T1WI and T2WI, coronal T2WI, and sagittal T1WI. (2) Contrast-enhanced scanning: CET1WI in transverse, coronal, and sagittal planes, of which fat suppression imaging was performed in one section. The parameters were as follows: T1WI [repetition time (TR) = 450 ms, echo time (TE) = 15 ms], T2WI (TR = 6000 ms, TE = 95 ms), FOV = 230 mm × 230 mm, matrix size = 512 × 168, flip angle = 90°, slice thickness = 5 mm, spacing between slices = 0.5 mm. The contrast agent, Gd-DTPA (Magnevist meglumine, Bayer Health Care Pharmaceuticals, Germany), was injected at a dose of 0.1 mmol/kg body weight (flow rate of 2.0 mL/s).

### Image analysis

All MR images were independently reviewed by two radiologists from our institution; both reviewers had at least 5 years of clinical experience in interpreting head and neck MRI images and were blinded to clinical data and survival outcomes.

The characteristics of the positive lymph nodes (pLNs) were examined on the initial MRIs to evaluate the lymph node (LN) burden. The lymphatic drainage regions involved retropharyngeal space and levels I–VII, and sub-levels (Ia vs. Ib, Va vs. Vb) were not considered separate. Matted LNs were counted as single when indistinguishable; the regions involved were recorded unilaterally and bilaterally. The numbers of pLNs and lymphatic drainage regions were recorded to calculate the RLND. The RLND was defined as the mean number of pLNs within the lymphatic drainage regions involved (eFig. 1), calculated using the following formula:

$${\rm{RLND}}\,{\rm{ = }}\,\frac{{number\,of\,pLNs}}{{number\,of\,lymphatic\,drainage\,regions\,involved}}$$

**Fig. 2:**
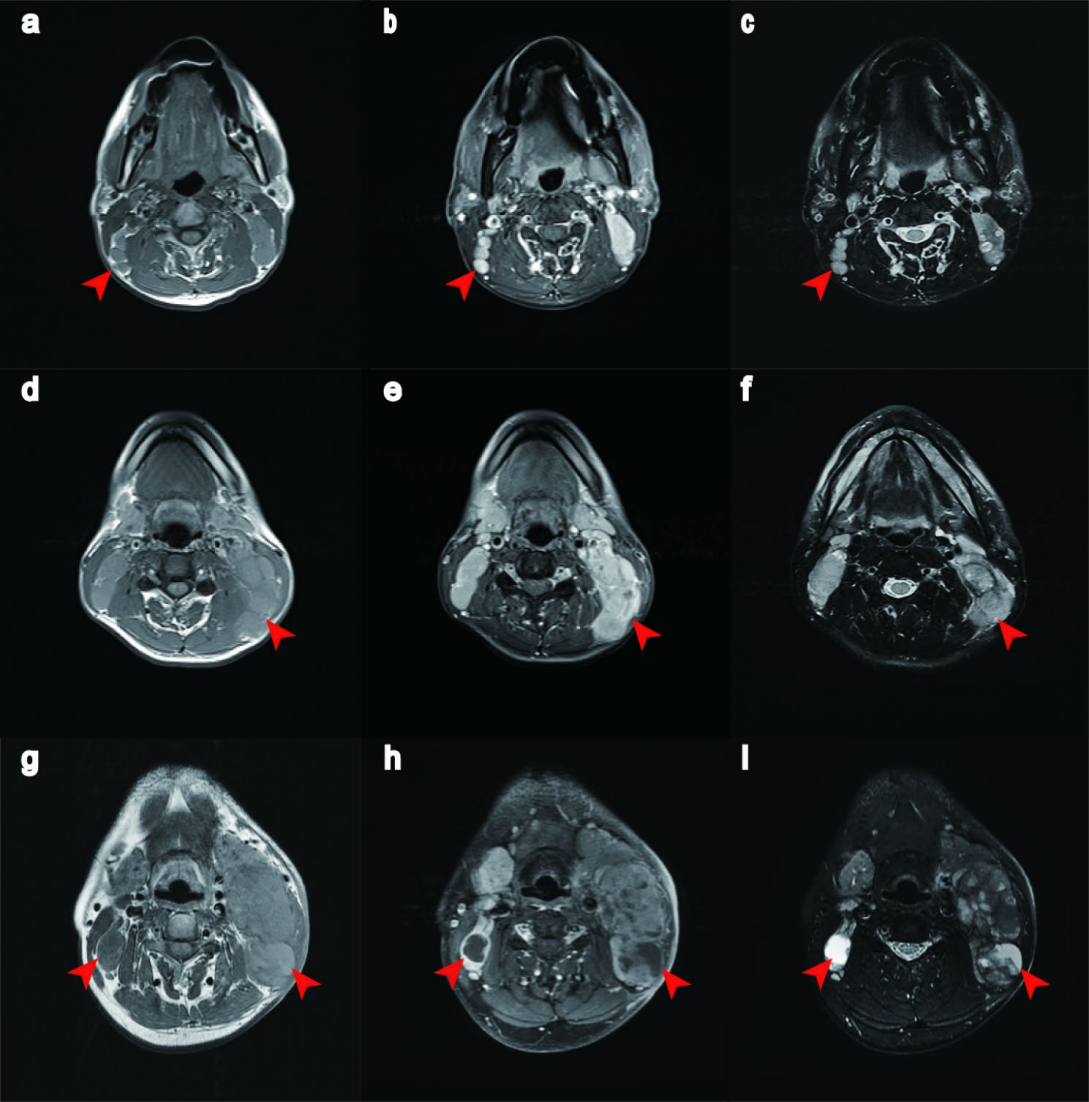
Representative MR images of different lymph node features (**a**) Axial T1-weighted, (**b**) axial contrast-enhanced T1-weighted, and (**c**) axial T2-weighted images in an NPC patient with NG (arrows). (**d**) Axial T1-weighted, (**e**) axial contrast-enhanced T1-weighted, (**f**) axial T2-weighted images in a patient with ENE (arrows). (**g**) Axial T1-weighted, (**h**) axial contrast-enhanced T1-weighted, (**i**) axial T2-weighted images in a patient with LNN (arrows)

The RLND for without pLN (N0) patients were identified as 0 according to the calculation formula. Other pLN characteristics documented on the MR images were the nodal maximum dimension (MD), laterality, nodal grouping (NG), lower-level involvement (LLI), lymph node necrosis (LNN), and extranodal extension (ENE) (Fig. 2). The diagnostic criteria for LN positivity: (1) minimal axial diameter (MID) ≥ 5 mm in the retropharyngeal region, ≥ 11 mm in the jugulodigastric region, or ≥ 10 mm for all other cervical nodes; (2) nodal grouping, the presence of three or more contiguous and confluent LNs, with a MID of at least 8 mm; (3) any LNs with necrosis; and (4) ENE [19].

The MD was measured in the plane with the largest diameter; NG was defined as the presence of three or more contiguous pLNs within the same region. LLI was defined as the presence of pLNs below the cricoid cartilage level, and ENE as infiltration into adjacent tissues, such as fat, muscles, or nerves. LNN was determined by the presence of a focal region of low signal intensity on contrast-enhanced T1-weighted images or high signal intensity on T2-weighted images, with or without an enhancing periphery. The degree of agreement between radiologists was assessed using the Cohen κ or intraclass correlation efficient (ICC) to estimate the inter-observer reliability for the MRI features considered.

### Treatment

All the study patients underwent IMRT. According to the National Comprehensive Cancer Network guidelines, patients with stage II disease received radiotherapy alone or with concurrent chemoradiotherapy. In contrast, patients in stage III-IVa received concurrent chemoradiotherapy with or without induction or adjuvant chemotherapy. Detailed information regarding the treatment is provided in Additional file 1.

### Endpoints

Our primary endpoint was the OS, calculated from the date of initial treatment to the most recent known date of survival or death from any cause. The secondary endpoint was the disease-free survival (DFS), calculated from the date of initial treatment to the first event occurring among relapse at any site, death from any cause, or date of the last disease-free follow-up visit.

### Statistical analysis

We conducted statistical analysis using the Student’s t-test for continuous variables and the χ2 test or Fisher’s exact test for categorical variables to determine any significant differences. The hazard ratios (HRs) for OS and DFS were calculated using univariate and multivariate Cox proportional hazard models. Nomograms were developed based on independent prognostic factors from the training cohort and subsequently validated in the validation cohort. We assessed the prognostic value of these nomograms through discrimination and calibration methods. We performed a calibration plot to visualize the agreement between predicted and observed survival curves. Additionally, we employed time-dependent receiver operating characteristic (ROC) curves and Harrell’s concordance index (C-index) to evaluate discrimination.

We evaluated the performance of the nomogram both with and without RLND using Harrell’s C-index to assess the impact of RLND on enhancing model prediction capabilities. A higher C-index suggested more accurate power for stratification. Moreover, we employed decision curve analysis (DCA) as a suitable method to examine the impact of RLND on NPC prognosis by evaluating alternative prognostic strategies. Recursive partitioning analysis (RPA) was applied to prognostic stratification. The survival rates of different risk groups were compared by the Kaplan-Meier (KM) curves with HR, 95% confidence intervals (CI), and log-rank *p*-values. All statistical analyses were performed using R software version 3.6.3 (R Core Team (2022). R: A language and environment for statistical computing. R Foundation for Statistical Computing, Vienna, Austria. https://www.r-project.org/; packages: “autoReg,” “survminer,” “rms,” “ROCit,” “nricens,” “broom,” “rpart,” and “survival’’). Statistical significance was defined as *p* < 0.05.

## Results

### Patient characteristics

**Fig. 3:**
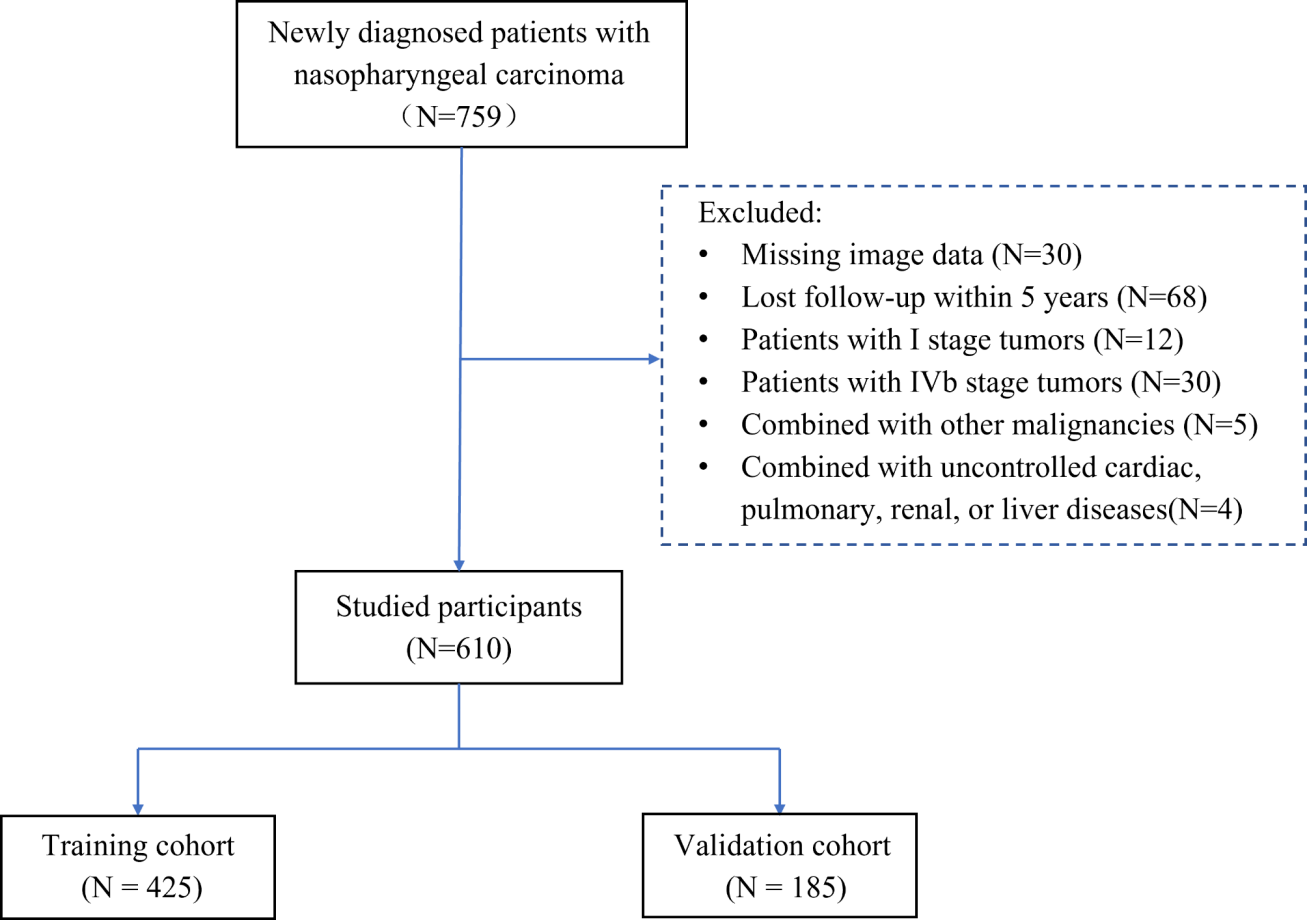
Flowchart illustrates patients’ selection

In total, 610 patients with NPC were included in this study. The sample sizes of the training and validation cohorts were 425 and 185, respectively; Fig. 3 illustrates a flowchart of the patient selection. The median age of the entire cohort was 46 years (interquartile range [IQR]: 37–53 years), and 74.3% of the patients were male. Overall, 21.1% had stage II disease, 34.3% stage III, and 44.6% stage IV_a_. The median nodal MD was 3.1 cm (IQR: 2–4.3 cm). Cervical lymph node metastases were unilateral in 35.4% of patients and bilateral in 39.2%. The incidence rates of NG, LLI, LNN, and ENE were 37.7%, 18.2%, 33.9%, and 46.6%, respectively. The patient characteristics and LN features are detailed in Table 1. The inter-observer agreement of the MRI feature assessments was good (kappa coefficients: 0.641–0.811; ICC: 0.836–0.878). Detailed information is provided in Additional file 2.

**Table 1:** Characteristics of the patients by cohort

Variables	Total cohort (n = 610)	Training cohort (n = 425)	Validation cohort (n = 185)	*p*
Age (years)	46 (37.0–53.0)	46 (37.0–53.0)	46 (38.0–56.0)	0.515
Gender				0.822
Male	453 (74.3)	314 (73.9)	139 (75.1)	
Female	157 (25.7)	111 (26.1)	46 (24.9)	
BMI (kg/m^2^)	22.1 (20.2–24.2)	22 (20.1–23.9)	22.4 (20.4–24.8)	0.135
Smoking status				0.567
Never	387 (63.4)	266 (62.6)	121 (65.4)	
Smoker	223 (36.6)	159 (37.4)	64 (34.6)	
WHO histologic type				0.692
I/II	69 (11.3)	50 (11.8)	19 (10.3)	
III	541 (88.7)	375 (88.2)	166 (89.7)	
T classification				0.324
T1	0 (0.0)	0 (0.0)	0 (0.0)	
T2	260 (42.6)	174 (40.9)	86 (46.5)	
T3	193 (31.6)	135 (31.8)	58 (31.4)	
T4	157 (25.7)	116 (27.3)	41 (22.2)	
N classification				0.549
N0	71 (11.6)	49 (11.5)	22 (11.9)	
N1	252 (41.3)	181 (42.6)	71 (38.4)	
N2	144 (23.6)	102 (24.0)	42 (22.7)	
N3	143 (23.4)	93 (21.9)	50 (27.0)	
Overall stage				0.956
II	129 (21.1)	91 (21.4)	38 (20.5)	
III	209 (34.3)	146 (34.4)	63 (34.1)	
IVa	272 (44.6)	188 (44.2)	84 (45.4)	
LLI				0.381
No	499 (81.8)	352 (82.8)	147 (79.5)	
Yes	111 (18.2)	73 (17.2)	38 (20.5)	
Laterality				0.917
Unilateral	216 (35.4)	150 (35.3)	66 (35.7)	
Bilateral	239 (39.2)	165 (38.8)	74 (40.0)	
None	155 (25.4)	110 (25.9)	45 (24.3)	
MD (cm)	3.1 (2.0-4.3)	3.1 (1.9–4.2)	3.4 (2.1–4.6)	0.087
RLND	1.2 (1.0-1.7)	1.2 (1.0-1.7)	1.2 (1.0-1.8)	0.482
NG				0.496
No	380 (62.3)	269 (63.3)	111 (60.0)	
Yes	230 (37.7)	156 (36.7)	74 (40.0)	
LNN				0.893
No	403 (66.1)	282 (66.4)	121 (65.4)	
Yes	207 (33.9)	143 (33.6)	64 (34.6)	
ENE				0.261
No	326 (53.4)	234 (55.1)	92 (49.7)	
Yes	284 (46.6)	191 (44.9)	93 (50.3)	
EBV DNA levels (copies/mL)				0.043
< 5000	270 (44.3)	200 (47.1)	70 (37.8)	
≥ 5000	340 (55.7)	225 (52.9)	115 (62.2)	
WBC (10^9^/L)	6.7 (5.5-8.0)	6.7 (5.6-8.0)	6.5 (5.4-8.0)	0.357
HGB (g/L)	139 (126–150)	138 (126–150)	140 (127–149)	0.933
PLT (10^9^/L)	272 (231–326)	273 (232–324)	269 (225–327)	0.731
NEUT (10^9^/L)	4.0 (3.2-5.0)	4.0 (3.2–5.1)	4.0 (3.1-5.0)	0.366
MONO (10^9^/L)	0.5 (0.4–0.6)	0.5 (0.4–0.6)	0.4 (0.4–0.6)	0.697
LYMPH (10^9^/L)	1.8 (1.4–2.2)	1.8 (1.4–2.2)	1.8 (1.5–2.2)	0.693
ALB (g/L)	41.2 ± 3.4	41.2 ± 3.6	41 ± 3.1	0.507
ALP (U/L)	71.5 (58–88)	72 (59–89)	69 (57–84)	0.221
LDH (U/L)	174 (151–206)	174 (151–208)	173 (150–203)	0.684
Treatment				0.507
RT alone	88 (14.4)	67 (15.8)	21 (11.4)	
CCRT	215 (35.2)	146 (34.4)	69 (37.3)	
CCRT + IC	264 (43.3)	181 (42.6)	83 (44.9)	
CCRT + AC	43 (7.0)	31 (7.3)	12 (6.5)	

Data are shown as means ± SD, median (IQR), or no. (%)

Abbreviations: SD, standard deviation; IQR, interquartile range; BMI, body mass index; LLI, lower levels involved; MD, nodal maximum dimension; RLND, regional lymph node density; NG, nodal grouping; LNN, lymph node necrosis; ENE, extranodal extension; EBV, Epstein–Barr virus; WBC, white blood cell count; HGB, hemoglobin; PLT, platelet count; NEUT, neutrophil count; MONO, monocyte count; LYMPH, lymphocyte count; ALB, albumin; ALP, alkaline phosphatase; LDH, lactate dehydrogenase; RT, radiotherapy; CCRT, concurrent chemoradiotherapy; IC, induction chemotherapy; AC, adjuvant chemotherapy

### Overall and Disease-Free Survival outcomes and predictors

At a median follow-up time of 73 months (IQR: 42–84 months) in the training cohort, 38.1% of the patients had disease progression, and 33.9% died. The 5-year OS and DFS rates were 68.7% and 64%, respectively. In the validation cohort, at a median follow-up time of 74 months (IQR: 56–85 months), 38.9% of the patients experienced disease progression, and 30.3% died. The 5-year OS and DFS rates were 75.1% and 64.8%, respectively.

Candidate variables for the prediction model were known risk factors and LN and demographic characteristics of clinical importance. The univariate analysis screened several factors associated with OS and DFS. The significant factors (*P* < 0.05) were included in the multivariate Cox regression model. Multivariate Cox proportional hazards models identified six variables that were independently associated with DFS and OS: RLND, age, T classification, lymphocyte count, LLI, and LNN. Smoking was independently associated exclusively with OS, as evidenced in Table 2 and Additional file 3.

**Table 2:** Results of multivariate analysis for OS and DFS

Characteristic	OS		DFS	
	HR (95% CI)	*p*-value	HR (95% CI)	*p*-value
Age	1.03 (1.01–1.04)	0.001	1.02 (1.01–1.04)	0.003
Smoking status				
Never	Reference			
Smoker	1.58 (1.09–2.30)	0.017		
T classification				
T1/2	Reference		Reference	
T3	1.70 (1.09–2.65)	0.020	1.52 (1.00-2.29)	0.048
T4	2.36 (1.49–3.73)	0.001	2.00 (1.30–3.09)	0.002
RLND	1.36 (1.03–1.80)	0.031	1.30 (1.00-1.69)	0.047
LLI				
No	Reference		Reference	
Yes	2.12 (1.39–3.25)	0.001	1.89 (1.26–2.83)	0.002
LNN				
No	Reference		Reference	
Yes	1.84 (1.26–2.67)	0.001	1.72 (1.20–2.45)	0.003
LYMPH	0.73 (0.56–0.96)	0.022	0.75 (0.58–0.96)	0.023

Abbreviations: OS, overall survival; DFS, disease-free survival; HR, hazard ratio; CI, confidence interval; RLND, regional lymph node density; LLI, lower levels involved; LNN, lymph node necrosis; LYMPH, lymphocyte count

### Prognostic Nomogram Development and Validation

**Fig. 4:**
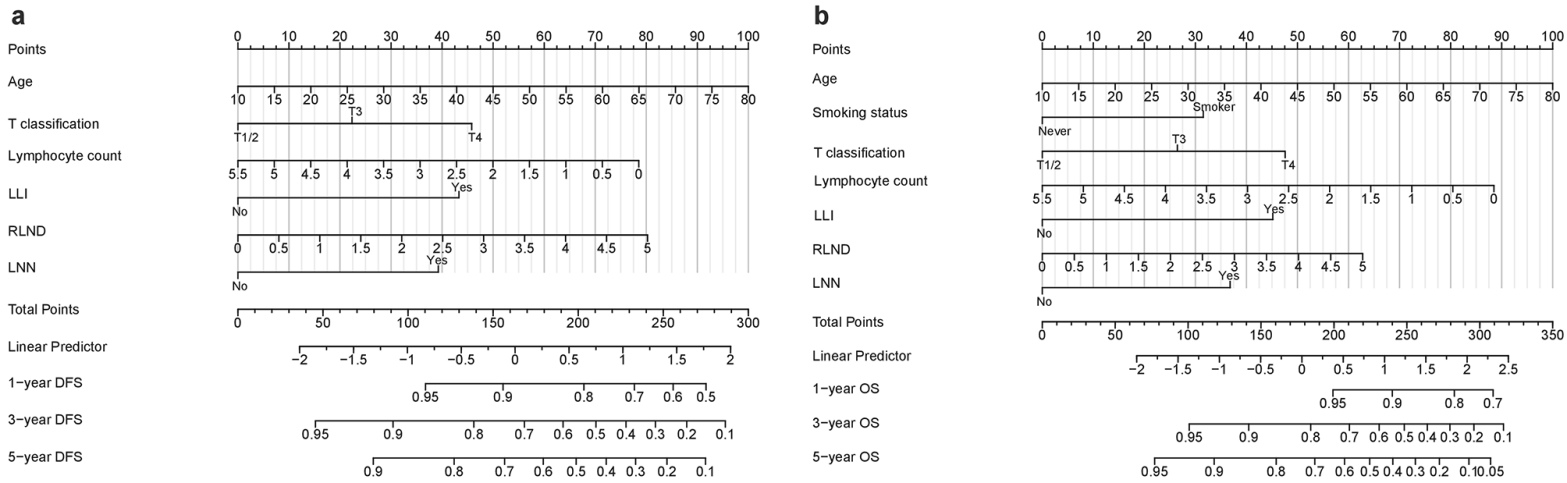
Nomograms of NPC patients without distant metastases for DFS (**a**) and OS (**b**)

Two nomograms were developed to provide quantitative and convenient tools to estimate the patient prognosis using the risk factors determined in the training cohort (Fig. 4). The C-index of the OS nomogram was 0.711 (95% CI: 0.700–0.722), which was greater than that of the TNM classification (0.680, 95% CI: 0.669–0.691). DFS nomogram model’s C-index (0.681, 95% CI: 0.670–0.692) was also greater than that of the TNM classification (0.669, 95% CI: 0.659–0.680). Similarly, The C-index of nomogram models (OS: 0.754, 95% CI: 0.738–0.771; DFS: 0.712, 95% CI: 0.700–0.727) was greater than that of the TNM classification (OS: 0.696, 95% CI: 0.678–0.714; DFS: 0.651, 95% CI: 0.635–0.667) in the validation cohort.

**Fig. 5:**
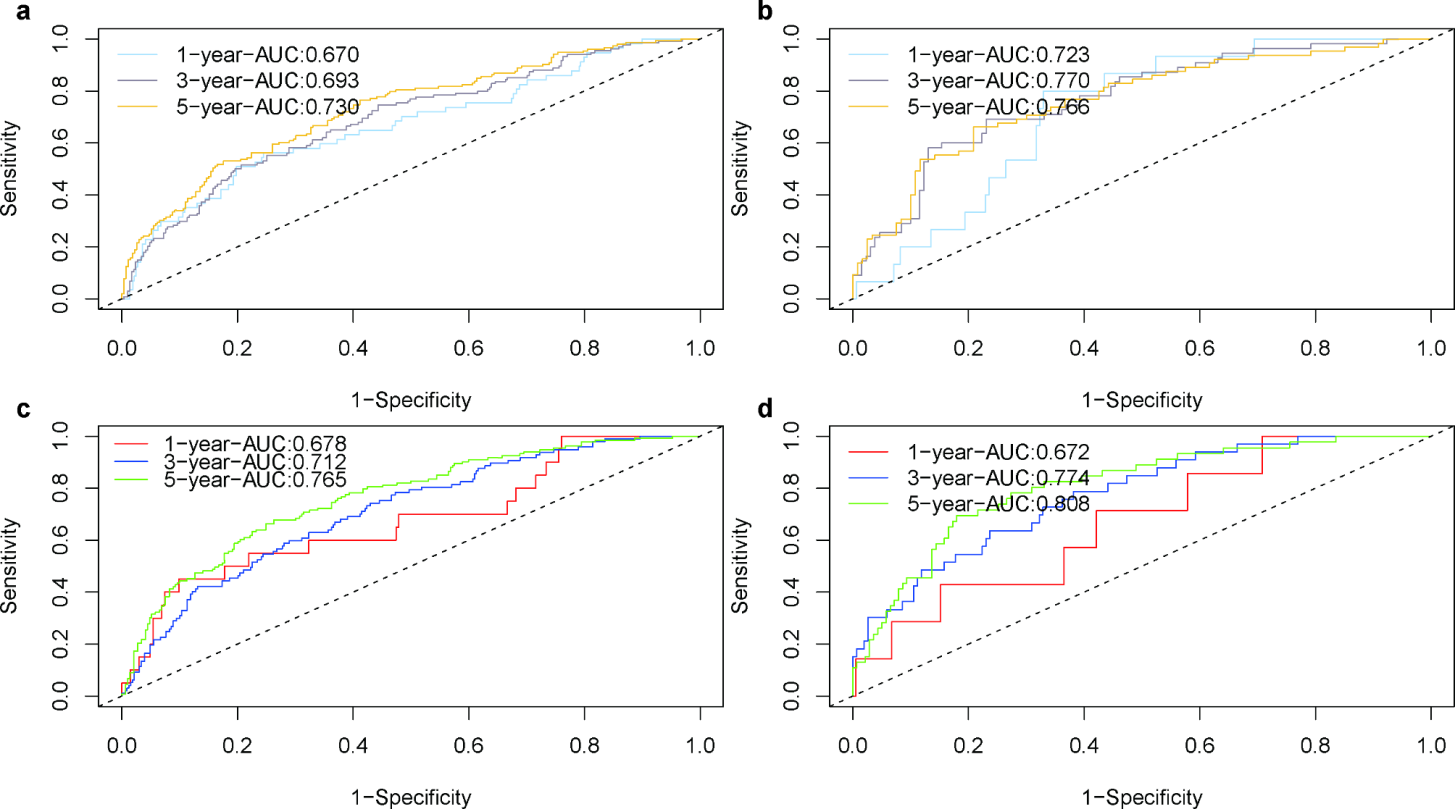
ROC curves for DFS (**a**, **b**) and OS (**c**, **d**) in training and validation cohort

Time-dependent ROC curves demonstrated the good discriminatory ability of the nomograms in the training and validation cohorts (Fig. 5). Furthermore, the calibration curves of the nomogram showed acceptable agreement between the prediction and actual observations (eFig. 2).

### Regional Lymph Node Density as a survival predictor

The two nomograms show that RLND was an effective common predictor for OS and DFS. To evaluate the impact of RLND on enhancing model prediction capabilities, we conducted a comparative analysis of the nomogram’s performance with and without RLND. In the training cohort, the C-index of RLND-based nomogram models (OS: 0.711; DFS: 0.681) surpassed that of nomogram models without RLND (OS: 0.686; DFS: 0.658). Similarly, in the validation cohort, RLND-based nomogram models (OS: 0.754; DFS: 0.712) outperformed the nomogram models without RLND (OS: 0.686; DFS: 0.644) in terms of C-index values.

The DCA curves suggested that both RLND and the combined nomogram had a certain clinical usefulness, and the combined nomogram provided higher clinical usefulness than RLND alone (eFig. 3).

### Optimal Nomogram score cutoff values for Outcome Prediction

**Fig. 6:**
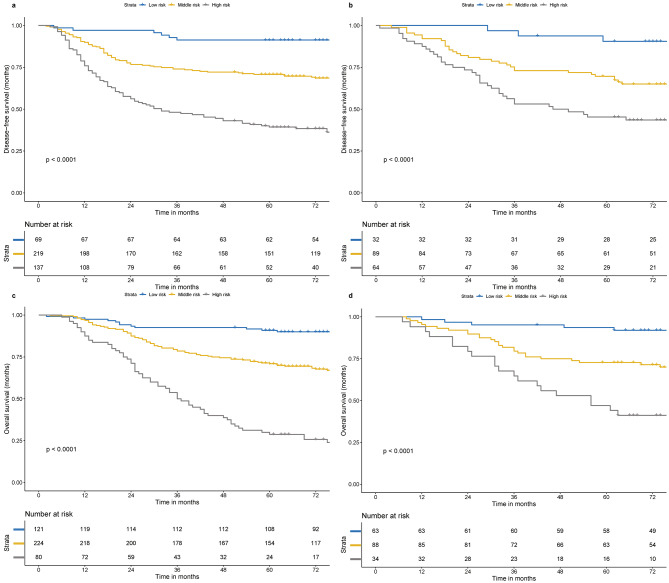
Kaplan–Meier curves for different risk groups on the DFS (**a**, **b**) and OS (**c**, **d**) in training and validation cohort

Given the nomograms’ effective predictive ability, we used them to conduct risk stratification, dividing the patients into a low-risk group (total scores ≤ 151.9), a middle-risk group (151.9 < total scores ≤ 220.3), and a high-risk group (total scores > 220.3) for low OS. We also determined the low-DFS low-risk group (total scores ≤ 121.0), the middle-risk group (121.0 < total scores ≤ 181.5), and the high-risk group (total scores > 181.5). The KM survival curves for OS and DFS were clearly separated between the three groups in the training and validation cohorts (*p* < 0.05; Fig. 6). Significantly worse outcomes were observed in high-risk patients in the training cohort, and a similar trend emerged in the validation cohort.

## Discussion

Several new NPC prognostic factors have been identified in the last few decades, including demographic characteristics, hematological biomarkers, and imaging features [13–16, 18, 20–22]. However, the most effective markers to estimate the prognosis of these patients remain to be determined. Our results showed that RLND is a robust prognostic factor; the RLND-based nomograms showed a reliable predictive performance superior to that of the TNM classification. A high degree of predictive accuracy was demonstrated in both the training and validation cohorts. RLND and RLND-based nomograms provided substantial clinical value in predicting the outcome of patients with NPC. Furthermore, RLND-based nomograms can effectively stratify patients into risk categories with significantly differing OS and DFS.

The extent of lymph node invasion is a mainstay prognostic factor in NPC. Lymph node involvement in this type of tumor progressively extends inferiorly within the neck [23]. Therefore, cervical lymph node laterality and the presence of pLNs beyond the caudal margin of the cricoid cartilage define the extent of lymph node invasion in the AJCC N classification [8]. However, it is difficult to quantify accurately the extent of invasion using only categorical factors. Therefore, we used the number of lymphatic drainage regions involved to quantify the lymph node invasion and the number of pLNs, a quantitative imaging indicator reported in recent studies. A large number of pLNs is connected with a dismal prognosis in NPC [18, 19]. We propose this new predictor by combining these two quantitative factors to obtain a robust indicator. RLND represents the lymph node metastasis density in all the regions involved since, with an equal number of these regions, a higher number of pLNs increases its value.

The prognostic value of metastatic lymph node features in NPC has been reported in several previous studies [14–16, 20, 24, 25], suggesting the presence of NG, ENE, LLI, LNN, and cervical lymph node laterality are correlated with worse outcomes in NPC. We identified RLND, LLI, and LNN, in particular, as independent prognostic factors. RLND is the only continuous variable among these indicators, providing distinct information in reflecting the lymph node burden in NPC.

The LN MD is an important indicator in the AJCC N classification [7]. However, our results showed that MD was not a distinct prognostic factor. Several reasons could account for this discrepancy. First, we measured the MD based on MR images rather than the clinical examination commonly used in the current staging manual. Second, the most reliable method to measure the MD using MRI remains to be determined. We measured only the dimensions of single or matted nodes to obtain the MD, whereas a previous study suggested measuring the dimensions of single, matted, or contiguous nodes [26]. Third, in the present study, the MD was included in the analysis as a continuous variable rather than converted into a categorical variable, as in previous studies. This difference could decrease the performance of MD measurements in predicting OS and DFS.

In addition, several demographic characteristics and hematological biomarkers have been proven effective prognostic factors for NPC. A high pretreatment lymphocyte count indicates a favorable prognosis, whereas smoking and older age are associated with a poor prognosis [27–29]. Our findings are in line with those of the studies mentioned above.

This study demonstrated the superiority of the RLND-based nomograms compared to the AJCC TNM classification in terms of survival prediction. However, further verification of the role of RLND in this model is needed. To address this, we developed nomograms with and without RLND. Our findings reveal that the inclusion of RLND elevates the C-index for the nomograms, affirming RLND’s pivotal role in augmenting the predictive capabilities of these models. Furthermore, to improve clinical practice and decision-making, we calculated the optimal cut-off values for the nomogram total score in OS (151.9 and 220.3) and DFS (121.0 and 181.5) and employed them for patient stratification. Consequently, these nomogram models successfully stratified stage II-IVa NPC patients into three distinct risk categories.

There are various limitations to this study. First, it was a single-center retrospective study, and a selection bias may have affected the findings; a multicenter prospective study is necessary to corroborate our findings. Second, our institution was located in an endemic area for NPC, and the results may not be generalized to non-endemic regions. Finally, counting manually all the pLNs and regions involved was time- and labor-intensive, possibly limiting the clinical application of RNLD; automatic counting using artificial intelligence models may be a practical solution to this problem.

## Conclusion

RLND is a robust prognostic factor in patients with NPC, especially when combined with other known factors. The RLND-based nomograms showed a reliable predictive performance, more accurate than the TNM classification.

### Electronic supplementary material

Below is the link to the electronic supplementary material.

Additional file 1

Additional file 2

Additional file 3

eFig. 1: Examples of regional lymph node density calculation

eFig. 2: Calibration plots for the nomograms. Calibration plots for OS in 1, 3, 5 years in training (a) and validation (b) cohort; Calibration plots for DFS in 1, 3, 5 years in training (c) and validation (d) cohort

eFig. 3: Decision curve analysis curves. The decision curve analysis of the nomogram and RLND for DFS in the training cohort (a) and validation cohort (b). The decision curve analysis of the nomogram and RLND for OS in the training cohort (c) and validation cohort (d)

## Data Availability

The datasets generated during and/or analyzed during the current study are available from the corresponding author upon reasonable request.

## Abbreviations

AJCC: American Joint Committee on Cancer
CI: Confidence interval
DCA: Decision curve analysis
DFS: Disease-free survival
EBV: Epstein-Barr virus
ENE: Extranodal extension
HR: Hazard ratio
IMRT: Intensity-modulated radiotherapy
IQR: Interquartile range
IRB: Institutional review board
KM: Kaplan-Meier
LLI: Lower-level involvement
LN: Lymph node
LNN: Lymph node necrosis
MD: Maximum dimension
NG: Nodal grouping
NPC: Nasopharyngeal carcinoma
NRI: Net reclassification index
OS: Overall survival
pLNs: Positive lymph nodes
RCS: Restricted cubic spline
RLND: Regional lymph node density
ROC: Receiver operating characteristic
TNM: Tumor-node-metastasis
