# Supplementary material for: Regional lymph node density-based nomogram predicts prognosis in nasopharyngeal carcinoma patients without distant metastases
Source: Cancer Imaging. 2023 Dec 15;23:123. doi: 10.1186/s40644-023-00641-z (PMC10724970; doi:10.1186/s40644-023-00641-z)
Supplement: Supplementary file 1 — Additional file 1 [file 40644_2023_641_MOESM1_ESM.docx]

***Detailed Information on Treatment***

All patients completed IMRT as planned. The delineation of target areas and organs at risk (OARs) was performed according to the International Commission on Radiation Units and Measurements (ICRU) Reports 50 and 62. Regarding the prescribed radiation doses, the primary gross tumor volume (GTVnx) was 68–75Gy/30-33f, cervical lymph node tumor volume (GTVnd) was 60-72Gy/30–33f, the clinical target volume with high risk (CTV1) was 60–64Gy/30–32f, and the low-risk clinical target volume (CTV2) was 54–58Gy/30–32f. All patients received radiotherapy once a day and 5 times per week. Concurrent chemotherapy was based on cisplatin (100 mg/m^2^ on days 1 to 3), and carboplatin was administered to some intolerant patients. Concurrent chemotherapy was administered every 3 weeks for 2–3 cycles. Induction chemotherapy or adjuvant chemotherapy was performed with TPF (docetaxel 60 mg/m^2^, day 1; cisplatin 60 mg/m^2^, day 1; 5-fluorouracil 600 mg/m^2^, continuous intravenous infusion for 120 hours), TP (docetaxel 75 mg/ m^2^, day 1; cisplatin 75 mg/m^2^, day 1), or PF (cisplatin 80 mg/m^2^, days 1 to 3; 5-fluorouracil 750 mg/m^2^, continuous intravenous infusion for 120 hours) regimens. Induction or adjuvant chemotherapy was administered every 3 weeks for 2–3 cycles.
