# Supplementary material for: Regional lymph node density-based nomogram predicts prognosis in nasopharyngeal carcinoma patients without distant metastases
Source: Cancer Imaging. 2023 Dec 15;23:123. doi: 10.1186/s40644-023-00641-z (PMC10724970; doi:10.1186/s40644-023-00641-z)
Supplement: Supplementary file 2 — Additional file 2 [file 40644_2023_641_MOESM2_ESM.docx]

| Results of inter-observer reliability for the obtained MRI features | | |  |  |
| --- | --- | --- | --- | --- |
| Variables |  | Cohen's Kappa coefficient | ICC | p |
| Laterality |  | 0.763 |  | 0.000 |
| RLND |  |  | 0.878 | 0.000 |
| MD |  |  | 0.836 | 0.000 |
| ENE |  | 0.811 |  | 0.000 |
| NG |  | 0.694 |  | 0.000 |
| LLI |  | 0.653 |  | 0.000 |
| LNN |  | 0.641 |  | 0.000 |
| Abbreviations: MRI, magnetic resonance imaging; ICC, intraclass correlation efficient; RLND, regional lymph node density; MD, nodal maximum dimension; ENE, extranodal extension; NG, nodal grouping; LLI, lower levels involved; LNN, lymph node necrosis. | | | | |
