# Supplementary material for: Regional lymph node density-based nomogram predicts prognosis in nasopharyngeal carcinoma patients without distant metastases
Source: Cancer Imaging. 2023 Dec 15;23:123. doi: 10.1186/s40644-023-00641-z (PMC10724970; doi:10.1186/s40644-023-00641-z)
Supplement: Supplementary file 3 — Additional file 3 [file 40644_2023_641_MOESM3_ESM.docx]

| Results of univariate analysis for OS and DFS | | | | | |
| --- | --- | --- | --- | --- | --- |
| Characteristic | OS |  |  | DFS |  |
|  | HR (95% CI) | p-value |  | HR (95% CI) | p-value |
| Sex |  |  |  |  |  |
| Male | Reference |  |  | Reference |  |
| Female | 0.58 (0.38-0.89) | 0.013 |  | 0.58 (0.39-0.86) | 0.007 |
| Age | 1.03 (1.01-1.04) | 0.000 |  | 1.02 (1.01-1.04) | 0.000 |
| BMI | 1.00 (0.95-1.05) | 0.943 |  | 1.00 (0.95-1.05) | 0.999 |
| WHO histologic type |  |  |  |  |  |
| I/II | Reference |  |  | Reference |  |
| III | 0.85 (0.52-1.38) | 0.508 |  | 0.89 (0.56-1.41) | 0.625 |
| Smoking status |  |  |  |  |  |
| Never | Reference |  |  | Reference |  |
| Smoker | 1.82 (1.31-2.52) | 0.000 |  | 1.72 (1.27-2.35) | 0.001 |
| T classification |  |  |  |  |  |
| T1/2 | Reference |  |  | Reference |  |
| T3 | 1.60 (1.06-2.44) | 0.027 |  | 1.49 (1.01-2.2) | 0.043 |
| T4 | 2.38 (1.59-3.56) | 0.000 |  | 2.04 (1.40-2.97) | 0.000 |
| N classification |  |  |  |  |  |
| N0 | Reference |  |  | Reference |  |
| N1 | 0.68 (0.37-1.26) | 0.217 |  | 0.72 (0.41-1.27) | 0.253 |
| N2 | 1.52 (0.83-2.80) | 0.173 |  | 1.65 (0.94-2.91) | 0.082 |
| N3 | 2.33 (1.29-4.21) | 0.005 |  | 2.16 (1.23-3.78) | 0.007 |
| Laterality |  |  |  |  |  |
| Unilateral | Reference |  |  | Reference |  |
| None | 0.71 (0.43-1.15) | 0.162 |  | 0.69 (0.44-1.08) | 0.107 |
| Bilateral | 1.59 (1.10-2.31) | 0.013 |  | 1.58 (1.12-2.23) | 0.010 |
| RLND | 1.41 (1.15-1.73) | 0.001 |  | 1.41 (1.17-1.71) | 0.000 |
| MD | 1.11 (1.03-1.20) | 0.008 |  | 1.09 (1.02-1.18) | 0.015 |
| ENE |  |  |  |  |  |
| No | Reference |  |  | Reference |  |
| Yes | 2.26 (1.61-3.16) | 0.000 |  | 2.31 (1.69-3.17) | 0.000 |
| NG |  |  |  |  |  |
| No | Reference |  |  | Reference |  |
| Yes | 2.04 (1.47-2.82) | 0.000 |  | 2.11 (1.55-2.87) | 0.000 |
| LLI |  |  |  |  |  |
| No | Reference |  |  | Reference |  |
| Yes | 2.42 (1.69-3.46) | 0.000 |  | 2.22 (1.57-3.14) | 0.000 |
| LNN |  |  |  |  |  |
| No | Reference |  |  | Reference |  |
| Yes | 2.46 (1.77-3.42) | 0.000 |  | 2.31 (1.69-3.14) | 0.000 |
| WBC | 0.99 (0.92-1.06) | 0.733 |  | 1.01 (0.94-1.07) | 0.881 |
| HGB | 1.01 (1.00-1.02) | 0.195 |  | 1.01 (1.00-1.02) | 0.185 |
| PLT | 1.00 (0.99-1.00) | 0.024 |  | 1.00 (1.00-1.00) | 0.040 |
| NEUT | 1.01 (0.93-1.09) | 0.857 |  | 1.03 (0.96-1.11) | 0.393 |
| LYMPH | 0.74 (0.56-0.97) | 0.031 |  | 0.76 (0.59-0.98) | 0.035 |
| MONO | 1.20 (0.99-1.44) | 0.063 |  | 1.19 (0.98-1.45) | 0.074 |
| ALB | 0.97 (0.92-1.02) | 0.198 |  | 0.98 (0.94-1.03) | 0.445 |
| LDH | 1.00 (1.00-1.01) | 0.000 |  | 1.00 (1.00-1.01) | 0.003 |
| ALP | 1.00 (0.99-1.01) | 0.754 |  | 1.00 (0.99-1.01) | 0.831 |
| EBV DNA (copies/mL) |  |  |  |  |  |
| <5000 | Reference |  |  | Reference |  |
| ≥5000 | 1.48 (1.06-2.06) | 0.022 |  | 1.38 (1.01-1.88) | 0.046 |
| Treatment |  |  |  |  |  |
| RT alone | Reference |  |  | Reference |  |
| CCRT | 1.31 (0.73-2.37) | 0.365 |  | 1.21 (0.71-2.07) | 0.478 |
| CCRT+IC | 1.99 (1.14-3.47) | 0.015 |  | 1.76 (1.07-2.90) | 0.026 |
| CCRT+AC | 2.27 (1.08-4.78) | 0.030 |  | 1.82 (0.90-3.69) | 0.095 |
| Abbreviations: OS, overall survival; DFS, disease-free survival; HR, hazard ratio; CI, confidence interval; BMI, body mass index; WHO, world health organization; LLI, lower levels involved; MD, nodal maximum dimension; RLND, regional lymph node density; NG, nodal grouping; LNN, lymph node necrosis; ENE, extranodal extension; EBV, Epstein–Barr virus; WBC, white blood cell count; HGB, hemoglobin; PLT, platelet count; NEUT, neutrophil count; MONO, monocyte count; LYMPH, lymphocyte count; ALB, albumin; ALP, alkaline phosphatase; LDH, lactate dehydrogenase; RT, radiotherapy; CCRT, concurrent chemoradiotherapy; IC, induction chemotherapy; AC, adjuvant chemotherapy. | | | | | |
