# Supplementary figures and images for: Regional lymph node density-based nomogram predicts prognosis in nasopharyngeal carcinoma patients without distant metastases
Source: Cancer Imaging. 2023 Dec 15;23:123. doi: 10.1186/s40644-023-00641-z (PMC10724970; doi:10.1186/s40644-023-00641-z)

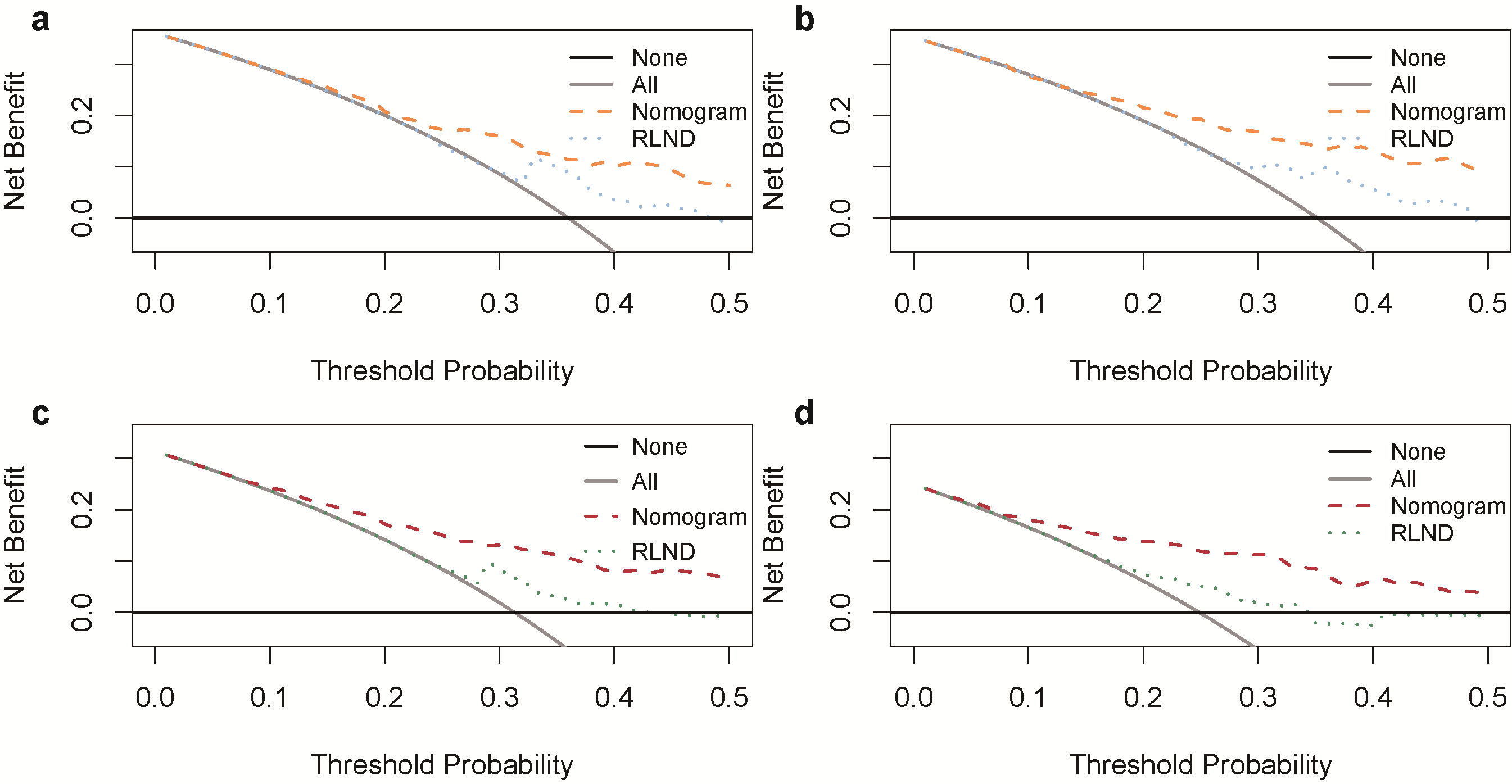

Supplement: Supplementary file 4 — eFig. 1: Examples of regional lymph node density calculation [file 40644_2023_641_MOESM4_ESM.png]

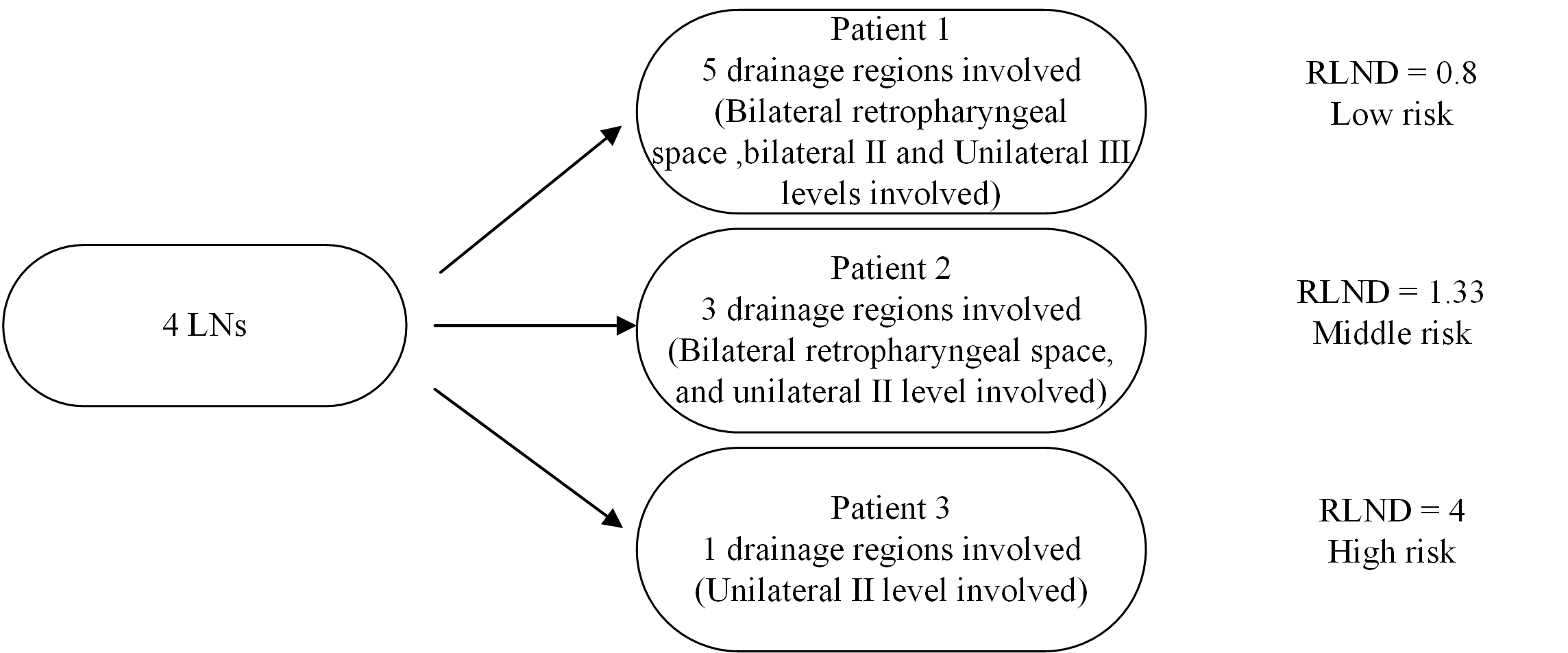

Supplement: Supplementary file 5 — eFig. 2: Calibration plots for the nomograms. Calibration plots for OS in 1, 3, 5 years in training (a) and validation (b) cohort; Calibration plots for DFS in 1, 3, 5 years in training (c) and validation (d) cohort [file 40644_2023_641_MOESM5_ESM.png]

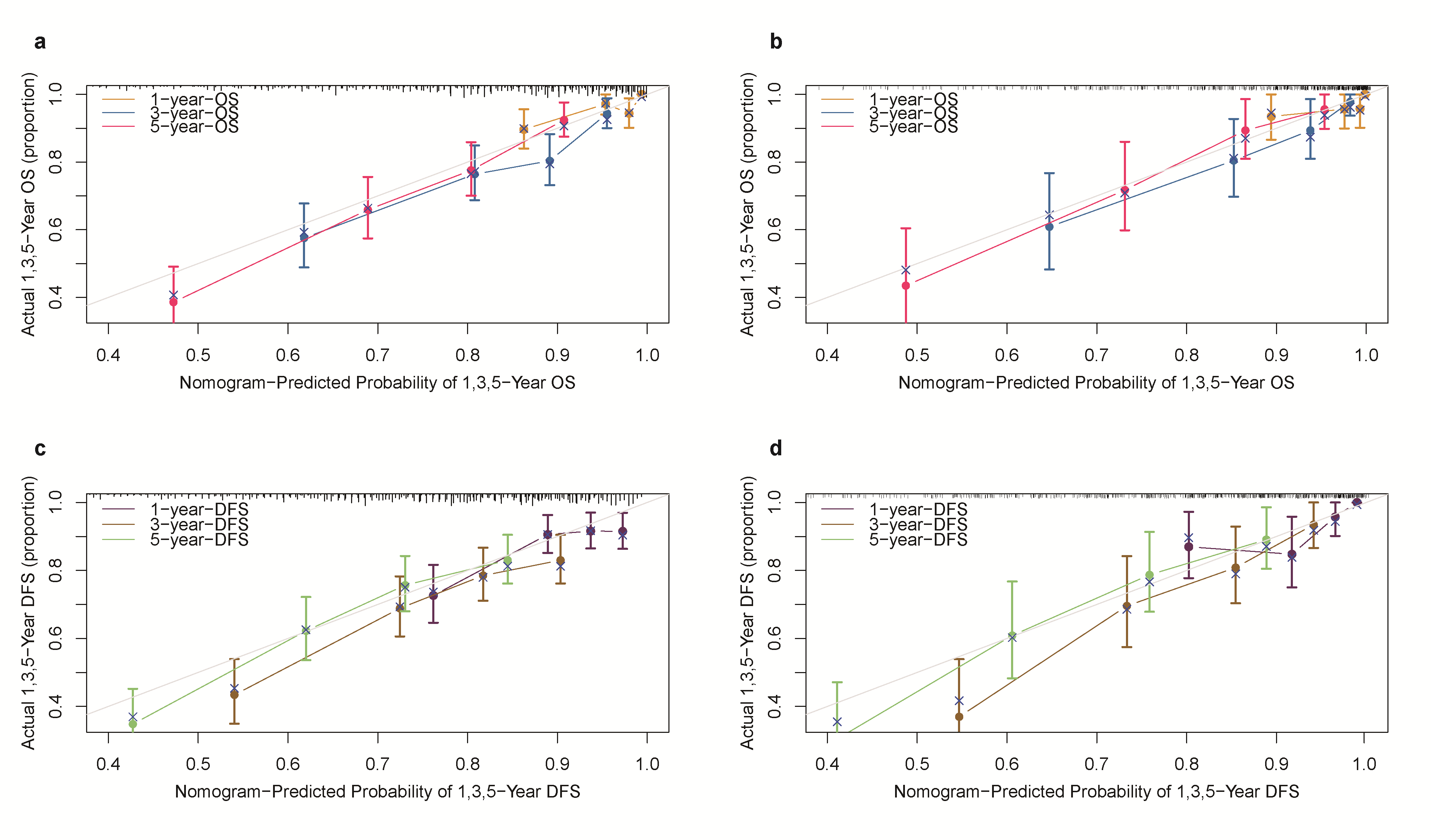

Supplement: Supplementary file 6 — eFig. 3: Decision curve analysis curves. The decision curve analysis of the nomogram and RLND for DFS in the training cohort (a) and validation cohort (b). The decision curve analysis of the nomogram and RLND for OS in the training cohort (c) and validation cohort (d) [file 40644_2023_641_MOESM6_ESM.png]
